# Transplanted Human Oligodendrocyte Progenitor Cells Restore Neurobehavioral Deficits in a Rat Model of Preterm White Matter Injury

**DOI:** 10.3389/fneur.2021.749244

**Published:** 2021-11-10

**Authors:** Xiaohua Wang, Jing Zang, Yinxiang Yang, Siliang Lu, Qian Guan, Dou Ye, Zhaoyan Wang, Haipeng Zhou, Ke Li, Qian Wang, Youjia Wu, Zuo Luan

**Affiliations:** ^1^The Second School of Clinical Medicine, Southern Medical University, Guangzhou, China; ^2^Department of Pediatrics, The Sixth Medical Center of PLA General Hospital, Beijing, China; ^3^Department of Pediatrics, Affiliated Hospital of Nantong University, Nantong, China

**Keywords:** preterm white matter injury, oligodendrocyte progenitor cell, myelination, neurobehavioral deficit, cell therapy

## Abstract

**Background:** Preterm white matter injury (PWMI) is a common brain injury and a leading cause of life-long neurological deficits in premature infants; however, no effective treatment is available yet. This study aimed to investigate the fate and effectiveness of transplanted human oligodendrocyte progenitor cells (hOPCs) in a rat model of PWMI.

**Methods:** Hypoxia-ischemia was induced in rats at postnatal day 3, and hOPCs (6 × 10^5^ cells/5 μL) were intracerebroventricularly transplanted at postnatal day 7. Neurobehavior was assessed 12 weeks post-transplant using the CatWalk test and Morris water maze test. Histological analyses, as well as immunohistochemical and transmission electron microscopy, were performed after transcardial perfusion.

**Results:** Transplanted hOPCs survived for 13 weeks in PWMI brains. They were widely distributed in the injured white matter, and migrated along the corpus callosum to the contralateral hemisphere. Notably, 82.77 ± 3.27% of transplanted cells differentiated into mature oligodendrocytes, which produced myelin around the axons. Transplantation of hOPCs increased the fluorescence intensity of myelin basic protein and the thickness of myelin sheaths as observed in immunostaining and transmission electron microscopy, while it reduced white matter atrophy at the level of gross morphology. With regard to neurobehavior, the CatWalk test revealed improved locomotor function and inter-paw coordination after transplantation, and the cognitive functions of hOPC-transplanted rats were restored as revealed by the Morris water maze test.

**Conclusions:** Myelin restoration through the transplantation of hOPCs led to neurobehavioral improvements in PWMI rats, suggesting that transplanting hOPCs may provide an effective and promising therapeutic strategy in children with PWMI.

## Introduction

Although advances in intensive care have led to improved survival of preterm infants, they have coincided with an increased incidence of premature brain injury. Preterm white matter injury (PWMI) is the most prevalent form of brain injury in premature infants and is the leading cause of cerebral palsy and other permanent neurological deficits (1). Currently, diffuse WMI characterized by global white matter hypomyelination, has become the most common type of PWMI observed in preterm infants (2). Diffuse WMI, as measured by diffusion tensor imaging, is associated with white matter atrophy, ventriculomegaly, and structural alterations of critical white matter tracts (3, 4).

The key pathological mechanism underlying PWMI is delayed or disturbed maturation of oligodendrocytes (OLs) and subsequent myelination (5). OL lineage cells develop through oligodendrocyte progenitor cells (OPCs), immature pre-myelinating OLs (pre-OLs), and finally mature into myelinated OLs (4, 6). OPCs and pre-OLs are predominant in the white matter from the gestational age of 23–32 weeks. As these immature OL lineage cells are highly sensitive to preterm-related insults, infants born at this gestational age with exposure to inflammation and/or hypoxia are at a high risk of impaired OL differentiation and myelination (5, 7). Furthermore, a lack of proper myelination during brain development will ultimately result in axonal degeneration and impaired brain connectivity (8).

Currently, no effective treatment is available for WMI in the developing brain. Stem/progenitor cell-based therapies are promising for treating neurological disorders, with potential neuroprotective and/or regenerative effects on injured brain cells. Neural stem cells (NSCs) and mesenchymal stem cells (MSCs) have been reported to improve neurobehavioral outcomes in some animal models of WMI (9, 10), however, they mainly play a neuroprotective role rather than regenerative replacement, because the differentiation rate of NSCs into OL is very low, and MSCs cannot differentiate into OL. Among stem cells, OPCs are considered to be the optimal cells for WMI treatment, as it has the potential to self-renew and differentiate into myelin-producing OLs (11, 12).

To date, there has been little published work on OPC-based therapies for PWMI. A limited number of studies have demonstrated that transplanted mouse-derived OPCs have neuroprotective effects in a rat model of periventricular leukomalacia (PVL, another type of PWMI), by stimulating endogenous NSC proliferation, inhibiting neuronal apoptosis, and improving cognitive impairment (13, 14). Evidently, there are species differences in myelin development and myelin regeneration between human-derived OPCs and mouse-derived OPCs (15), and human OPCs are superior when it comes to translatability for clinical trials. In 2018, a study by Tae-Kyun et al. showed that human-derived F3.olig2 OPCs (established from an immortalized human NSC line) restored neurobehavioral functions by preventing axonal demyelination in a rat model of PVL (16). However, this study of human OPC treatment for PWMI described a possible neuroprotective effect of OPCs within 5 weeks, but not a regenerative effect. Thus, our study was designed to observe the migration and differentiation capacity of hOPCs during a 13-week observation period and to determine whether transplanted hOPCs could regenerate myelin and improve neurobehavioral outcomes in a rat model of PWMI.

## Materials and Methods

### Animal Model of PWMI

This study was approved by the Animal Ethical and Welfare Committee of the Chinese PLA General Hospital (Beijing, China) (Ethics No. SCXK-2012-0001). The number and suffering of the animals were minimized as much as possible. The day of birth was defined as postnatal day (P) 0. Hypoxia-ischemia (HI) was induced in rats at P3 as previously described (17, 18). Briefly, 38 Sprague-Dawley pups of either sex from three litters were anesthetized by placing them on ice for 10 min, and the right common carotid artery was isolated and permanently occluded by electrocoagulation. Pups were allowed to recover for 30–60 min and then were placed in a container perfused with a humidified gas mixture (6% oxygen in nitrogen) at 37°C for 90 min. Thirty surviving pups were randomized into one of two groups: PWMI or PWMI+hOPCs (*n* = 15 per group). Animals in the control group (*n* = 10) underwent surgery without arterial occlusion or subsequent hypoxia.

### Preparation of hOPCs

The hOPCs were prepared and provided by the Pediatric Laboratory at the Sixth Medical Center of the Chinese PLA General Hospital, according to the guidelines approved by the Hospital's Ethics Committee (Ethics No. 2015013). Briefly, hNSCs were isolated from the cortex of human embryos aged 10–13 weeks and amplified in the NSC medium. To generate hOPCs, hNSCs were cultured for 10 days to form neurospheres, which were dissociated into single cells and induced to differentiate in the differentiation medium (19). The purity of the hOPCs was 80–90%, as confirmed in our previous study (20). Before transplantation surgery, hOPCs were stained with specific markers such as PDGFR-α, A2B5, NG2, and O4 (see Supplementary Figure 1) and then resuspended in 5 μL of PBS to make a single-cell suspension containing 6 × 10^5^ cells.

### Transplantation of hOPCs

Four days after HI insult, pups in the PWMI+hOPCs group were intracerebroventricularly transplanted with hOPCs. Briefly, pups were placed in a stereotaxic device under isoflurane anesthesia (induction at 2.5% and then 1% for maintenance). An incision was made to the skin, and a needle was inserted through the skull at the following coordinates (from Bregma): anteroposterior, −0.5 mm; mediolateral, −1.5 mm; and dorsoventral, −2.0 ~ −2.5 mm. Pups were injected with 6 × 10^5^ cells (5 μL) over the course of 10 min. The syringe was left *in situ* for 5 min and then gradually withdrawn. The pups were placed on a heated blanket to recover before being returned to the dam. Animals in the PWMI group received an injection of 5 μL PBS at the same coordinates. All groups received cyclosporine A (10 mg/kg/day; Sandimmun, Novartis) intraperitoneally for 4 weeks starting 3 days before transplantation, followed by 100 μg/mL in the drinking water until the perfusion.

### Neurobehavioral Tests

Twelve weeks after transplantation, the rats were subjected to behavioral tests: the CatWalk test and Morris water maze test. These tests were performed between 8 am and 11 am by a well-trained investigator and an assistant who were blinded to the experimental groups.

### CatWalk Test

The CatWalk test was used to objectively assess walking ability, and was carried out with an automated computer-assisted system (Noldus Information Technology, The Netherlands) (21, 22). An enclosed glass walkway was illuminated with fluorescent light coming from the side. The light reflected by the rats' paws as they contacted the walkway was captured by a high-speed video camera and then transformed into a digital image. Before the test, rats were trained to cross the glass walkway toward their home cage. The test was conducted in a dark, silent room. The runs with accepted step cycles were analyzed for each rat. If a rat failed to complete a run within 5 s, walked backward, or reared on the glass floor, it was given an additional run, and an alternative motivator such as food reward was used. Two rats in PWMI group and three rats in PWMI+hOPCs group were excluded because they failed to complete the run. The videos were analyzed using CatWalk XT 10.6, and footprints were automatically or manually labeled as right fore (RF), right hind (RH), left fore (LF), and left hind (LH) paws. After identification of individual footprints, data including individual paw statistics, comparative paw statistics, interlimb coordination, and temporal parameters were all automatedly analyzed and exported.

### Morris Water Maze Test

The Morris water maze test was performed to evaluate spatial learning and memory, as previously described (13, 23). Briefly, this task was performed in a circular device with a diameter of 150 cm and a height of 50 cm and was filled with water (30 cm in depth, 22 ± 2°C). A black hidden platform (12 cm in diameter) was placed in Quadrant 2, ~1.5 cm beneath the water surface. Rats in their home cage were placed in the testing room for at least 1 h before the test to minimize the effects of stress on behavioral outcomes. For the navigation test, rats underwent four trials daily with different drop locations, over five consecutive days. Each trial ended when the rat rested on the platform for 3 s. If a rat could not find the platform within 60 s, it was led to the platform and allowed to rest for 10 s. The space probe trial, with the platform removed, took place on the sixth day over 60 s. Data were analyzed using the Morris water maze system (Labmaze V3.0; Zhongshidichuang Science and Technology Development Co., Ltd, Beijing, China). The latency to find the platform, number of platform crossings, number of platform quadrant crossings, and time spent in the platform quadrant were recorded.

### Tissue Preparation and Histological Assessment

Thirteen weeks after transplantation, the rats were deeply anesthetized with 10% chloral hydrate and transcardially perfused with 0.9% saline followed by 4% paraformaldehyde (PFA). Brains were removed and post-fixed in 4% PFA at 4°C for 24 h, followed by cryoprotection in a graded series of sucrose (15% and 30%). Coronal sections were cut at 8 μm using a freezing microtome (Cm1850; Leica, Germany) and stored at −80°C. Brain sections (2.04, 1.32, 0.12, −0.84, −1.92, −3.00, and −5.40 mm from Bregma) were stained with hematoxylin and eosin (HE) and Luxol fast blue (LFB), as previously described (24, 25). All stained sections were scanned on a computer using an Axio scan (Z1 system; Zeiss, Germany).

To evaluate cerebral atrophy, on each section with HE staining, the areas of both hemispheres were measured by ImageJ software (NIH, USA), and the volume was calculated by multiplying the appropriate area by the section interval thickness. To minimize errors associated with tissue processing, the relative volume of cerebral atrophy was determined using the following formula: (contralateral cerebral volume—ipsilateral cerebral volume)/contralateral cerebral volume (26).

To evaluate white matter atrophy, in each section with LFB staining, the white matter areas (including the corpus callosum, external capsule, internal capsule, and striatum fiber bundles) within the ipsilateral and contralateral hemispheres were measured. The volume was calculated according to the same principle as above, and the relative volume of white matter atrophy was determined as follows: (contralateral white matter volume—ipsilateral white matter volume)/contralateral white matter volume.

### Immunohistochemistry and Imaging

Frozen sections were blocked with 2% BSA/0.3% Triton X-100 in PBS for 1 h and incubated with the following primary antibodies overnight at 4°C: anti-hN (1:300, mouse monoclonal, Millipore) and anti-stem121 (1:500, mouse monoclonal, TaKaRa) for the detection of transplanted human cells; anti-GFAP (1:1,000, rabbit polyclonal, Abcam) for the detection of astrocytes; anti-Olig2 (1:200, rabbit polyclonal, Millipore) for the detection of oligodendrocytes; anti-NF (1:500, rabbit polyclonal, BioLegend) for axon staining; and anti-myelin basic protein (MBP) (1:100, rabbit polyclonal, Abcam) for myelin staining. Subsequently, the sections were washed in PBS and incubated in the appropriate secondary antibodies conjugated with Alexa Fluor-488 or−594 for 1 h at 37°C. Images were acquired immediately after staining using a fluorescence microscope (Olympus IX51, Japan). To quantify the differentiation capacity of hOPCs into Olig2^+^ oligodendrocytes, the proportion of hN^+^ cells that were also Olig2^+^ was determined in five animals. Images were taken from three 20 × objective fields per section and from three sections (2.04, 0.12, and −5.40 mm from Bregma) per animal, namely, nine sampling regions per animal. To quantify the differentiation capacity of hOPCs into MBP^+^ oligodendrocytes or GFAP^+^ astrocytes, the area fraction of stem121^+^/MBP^+^ or stem121^+^/GFAP^+^ to stem121^+^ was measured using ImageJ in five animals. Images were taken from nine 40 × objective fields of each animal according to the same principle as described above. To ascertain whether processes of human-derived cells were wrapped around host axons, a three-dimensional reconstruction by Z-stack scanning through regions of interest was acquired with a confocal microscope (Olympus FV300, Japan).

Immunostaining of MBP (1:100, rat monoclonal, Abcam) was performed to determine the integrity of the host myelin. Alexa Fluor-488 conjugated donkey anti-rat IgG (1:200, Abcam, UK) was used as the secondary antibody. To detect the MBP intensity of the ipsilateral cingulate gyrus and external capsule, region-specific images were taken from three adjacent sections (~0.12 mm from Bregma) in each animal, and the green intensity was analyzed using ImageJ.

### Transmission Electron Microscopy (TEM)

The corpus callosum (~1 × 1 × 1 mm), adjacent to the transplant site, was isolated from the forebrain and fixed at 4°C in a solution containing 2.5% glutaraldehyde and 2% PFA. The samples were subsequently embedded in epoxy resin, sliced using an ultrathin microtome, and stained with uranium acetate and lead citrate. Ultrathin sections were observed under a transmission electron microscope and photographed for further analysis. These procedures were mostly performed at the Electron Microscopy Center of Tsinghua University. TEM images were analyzed for g-ratio using Image-Pro Plus 6.0 (Media Cybernetics, USA). G-ratio was defined as the ratio of the diameter of an axon to the diameter of the axon plus its myelin sheath (24). Quantification was performed in 50 randomly chosen myelinated axons at 6,000× magnification for each animal (*n* = 4 per group).

### Statistical Analysis

Data are presented as mean ± SEM, unless stated otherwise. Continuous variables of the Morris water maze test were analyzed by repeated measures two-way analysis of variance (ANOVA), followed by Fisher's least significant difference (LSD) *post hoc* test when appropriate. Gait analysis data were compared using the Kruskal–Wallis non-parametric test, followed by a Mann-Whitney *U*-test when the results were significant. Other data were analyzed using one-way ANOVA. The statistical significance level was set at *P* ≤ 0.05. Data analysis and statistical inference were performed using the SPSS 25.0 (IBM, Armonk, NY).

## Results

### Migration and Differentiation of Transplanted hOPCs in PWMI Rats

**Figure 1:**
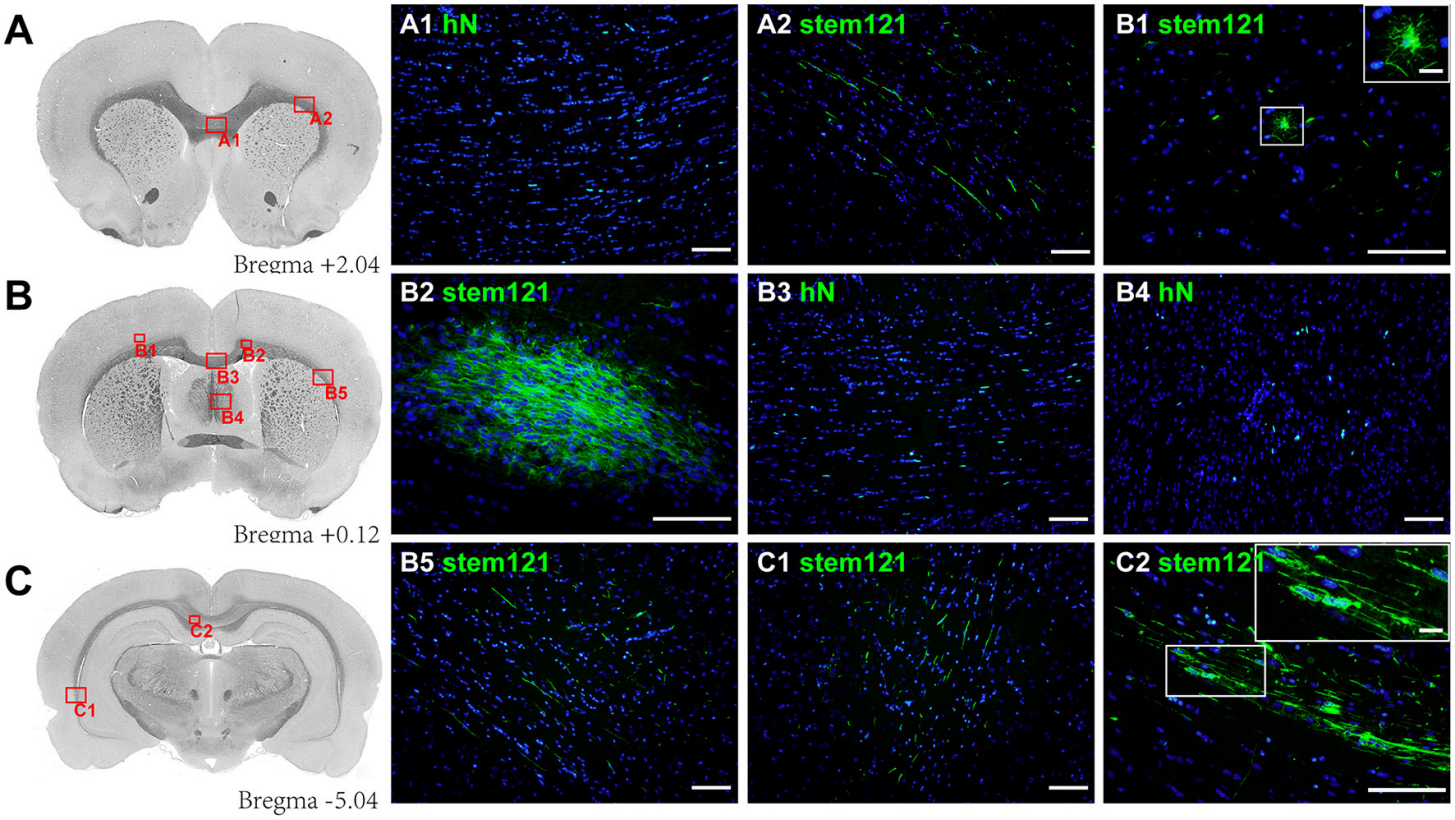
Migration of transplanted hOPCs. **(A–C)** Stem121^+^/hN^+^ cells were widely distributed in the injured white matter and migrated to the contralateral brain in PWMI rats at 13 weeks after transplantation. Stem121^+^ cells in the lower layers of the cortex were round with extensive and radial processes (B1), while cells in the corpus callosum were spindle-shaped with long parallel processes (C2). All coronal sections were counterstained with DAPI. Scale bar represents 100 μm in (A1-C2) and 15 μm in zoomed images of B1 and C2.

At 13 weeks post-transplantation, stem121^+^/hN^+^ cells were widely distributed in injured white matter areas and migrated through the corpus callosum to the contralateral cingulate gyrus and external capsule. Scattered cells were also found in the lower layers of the cortex and fimbria of the hippocampus (Figure 1). The hN^+^ cells were the most highly concentrated near the transplant site (522.67 ± 145.55), followed by the section at −5.40 mm from Bregma (387.11 ± 140.41) and the section at 2.04 mm from Bregma (270.00 ± 38.00); however, there was no statistical difference in hN^+^ cell counts between these three sections [*F*_(2,6)_ = 4.830, *P* = 0.056; one-way ANOVA]. Interestingly, stem121^+^ cells in the lower layers of the cortex were round with radial processes (Figure 1B1), consistent with type I morphology, while cells in the corpus callosum and external capsule were spindle-shaped with long parallel processes (Figure 1C2), consistent with type II/III morphology (27).

**Figure 2:**
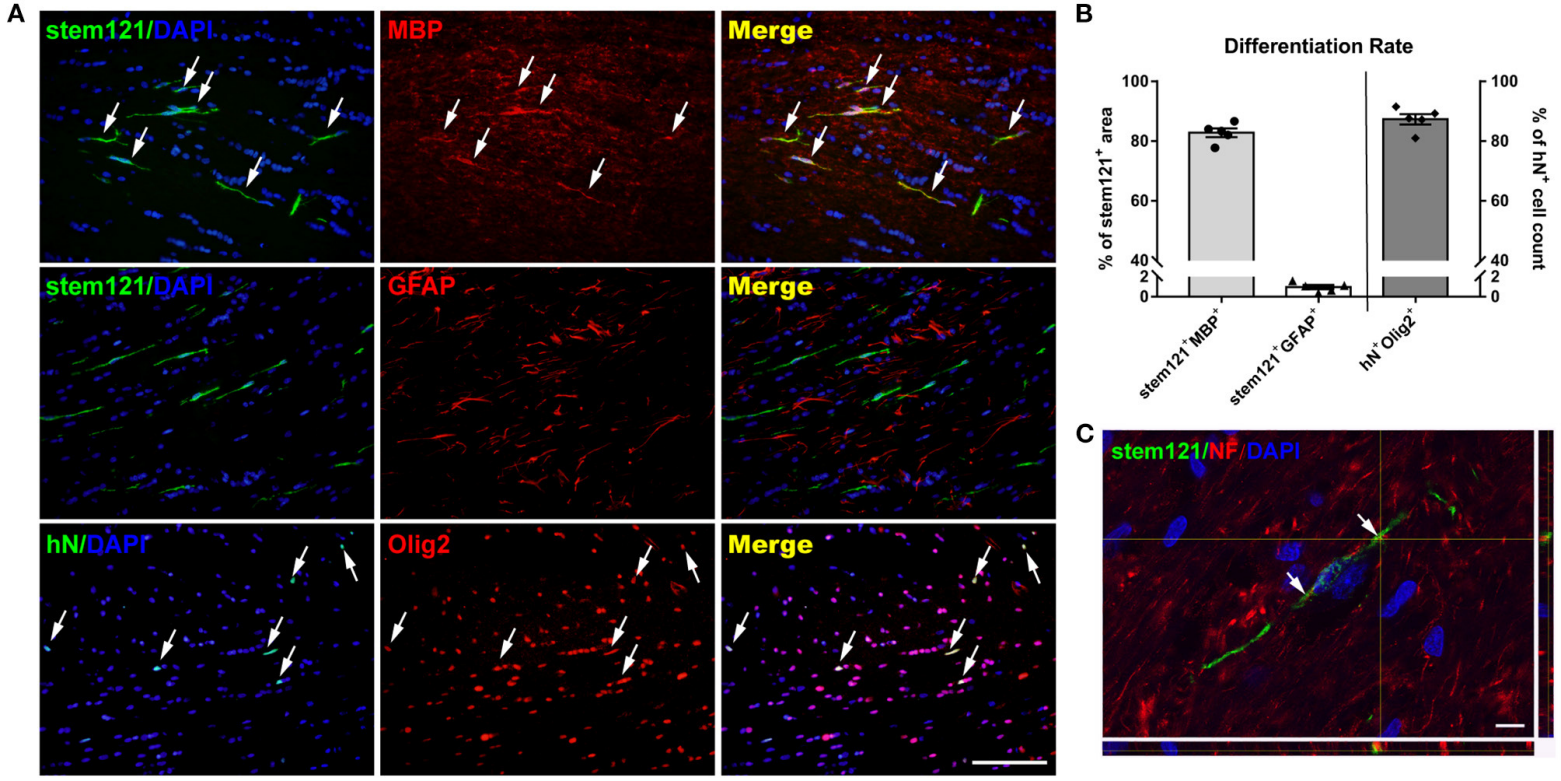
Differentiation of transplanted hOPCs. **(A)** Representative images of hOPCs differentiation at 13 weeks post-transplantation. **(B)** More than 80% of transplanted cells co-expressed Olig2 and MBP, and little GFAP co-staining was observed in stem121^+^ cells (*n* = 5 per group; three sections per rat, with three sites randomly sampled per section). **(C)** Confocal images showed the ensheathment of axons by stem121^+^ OLs. Scale bar represents 50 μm in **(A)** and 10 μm in **(C)**. Data are presented as mean ± SEM.

To identify and quantify the differentiation of transplanted cells, double immunostaining was performed. The majority (87.29 ± 1.75%) of hN^+^ cells co-expressed Olig2, and the area fraction of stem121^+^MBP^+^/stem121^+^ was up to 82.77 ± 3.27%, while the area fraction of stem121^+^GFAP^+^/stem121^+^ was 0.97 ± 0.21% (Figures 2A,B), suggesting that most transplanted cells matured to be MBP-producing OLs in injured white matter, and only a few cells differentiated into astrocytes. Importantly, reconstructed confocal images showed a close, ensheathment-like position of NF^+^ stem121^−^ host axons with stem121^+^ cells differentiated from hOPCs (Figure 2C).

### hOPCs Restored Myelin Integrity of PWMI Rats

**Figure 3:**
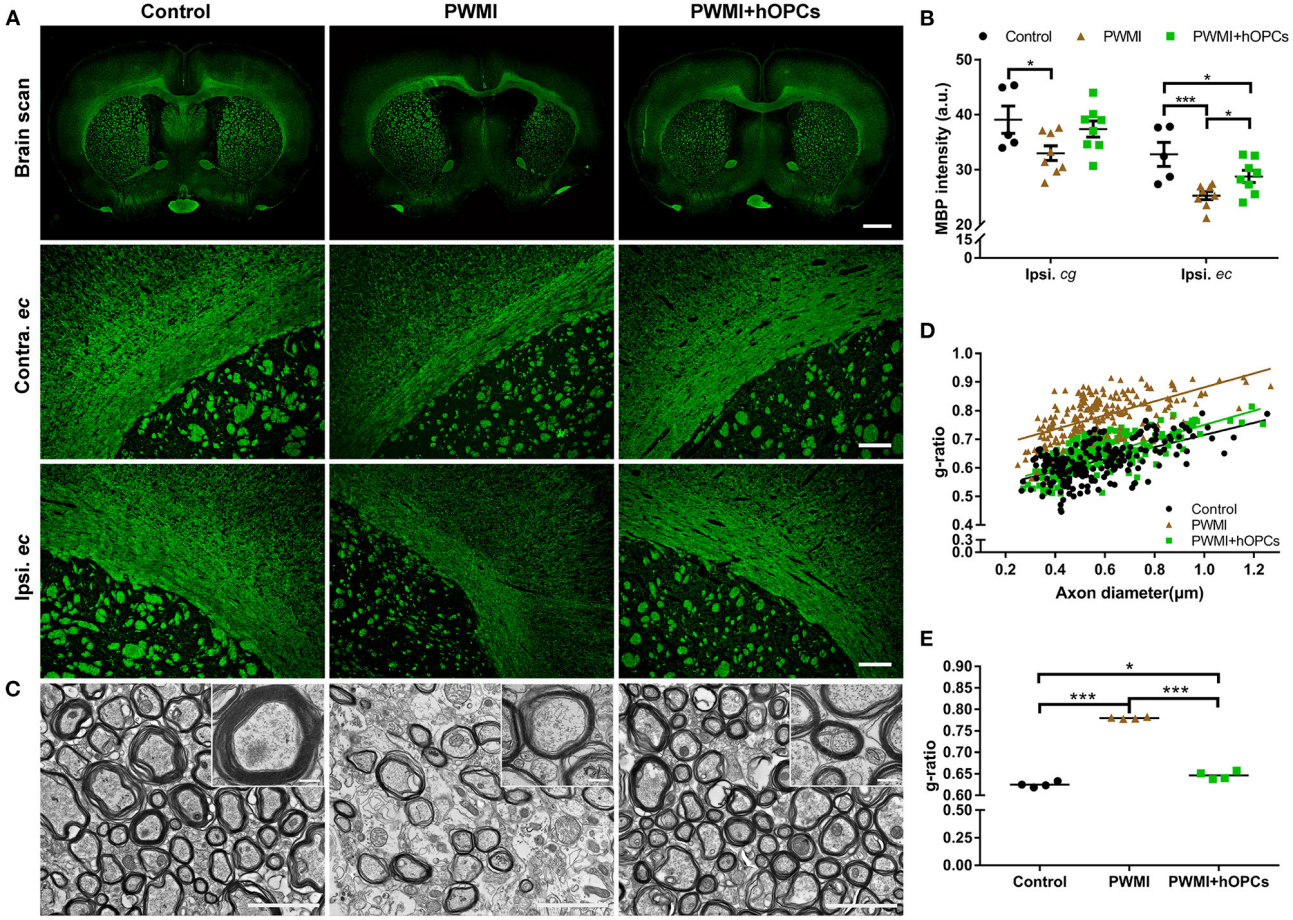
Immunohistochemical and TEM analysis of myelin. **(A)** Representative images of MBP intensity in both hemispheres and external capsules at 13 weeks post-transplantation. Scale bar represents 2,000 μm in the top row and 200 μm in the two lower rows. **(B)** Quantification of MBP intensity in the ipsilateral cingulate gyrus (*cg*) and external capsule (*ec*) (*n* = 5 for control, *n* = 8 for PWMI and PWMI+hOPCs; three sections per rat; one-way ANOVA with LSD for multiple comparisons). **(C)** Representative TEM images of myelin sheath in the corpus callosum. Scale bar represents 2 μm in **(C)** and 200 nm for the insert pictures. **(D,E)** G-ratios of myelinated axons in the corpus callosum of the control, PWMI, and PWMI+hOPCs groups (*n* = 4 per group, one-way ANOVA with LSD for multiple comparisons). Data are presented as mean ± SEM. ^*^P < 0.05, ^***^P < 0.001.

Compared with the control rats, HI-injured rats displayed decreased MBP intensity in both hemispheres 13 weeks post-transplantation, and the intensity in the ipsilateral hemisphere was lower than that in the contralateral hemisphere, especially in the external capsule (Figure 3A). Quantitative analysis revealed that MBP intensity in the ipsilateral cingulate gyrus and external capsule was significantly lower in HI-injured rats than in control rats [cingulate gyrus: *F*_(2,18)_ = 3.550, *P* = 0.050, PWMI vs. control, *P* = 0.025; external capsule: *F*_(2,18)_ = 8.145, *P* = 0.003, PWMI vs. control, *P* = 0.001; one-way ANOVA with LSD for multiple comparisons]. MBP in the cingulate gyrus of the PWMI+hOPCs group was comparable to that in both the control group and the PWMI group, while MBP expression in the external capsule of the PWMI+hOPCs group was more intense than that of the PWMI group (*P* = 0.047; LSD), but still lower than that of the control group (*P* = 0.046; LSD) (Figure 3B).

We next used TEM to visualize the ultrastructure of the demyelinated areas. In control animals, a large amount of thick and compact myelin sheaths was observed in the corpus callosum. In comparison, the myelin sheath was sparse in the PWMI rats, with a thin and disrupted structure. The hOPC-transplanted rats exhibited evidence of structural restoration, as observed in the density and thickness of the myelin sheath, but the restored myelin was thinner than the normal myelin sheath (Figure 3C). These findings were confirmed by the quantification of the g-ratio (0.625 ± 0.006 in the control group, 0.780 ± 0.002 in the PWMI group, and 0.647 ± 0.009 in the PWMI+hOPCs group) [*F*_(2,9)_ = 4.622, *P* < 0.001, PWMI vs. control, *P* < 0.001, PWMI+hOPCs vs. PWMI, *P* < 0.001, PWMI+hOPCs vs. control, *P* = 0.027; one-way ANOVA with LSD for multiple comparisons; Figures 3D,E]. Taken together, these results demonstrate that transplanted hOPCs restored the integrity of the myelin sheath in PWMI rats.

### hOPCs Improved Neuropathological Outcomes of PWMI Rats

**Figure 4:**
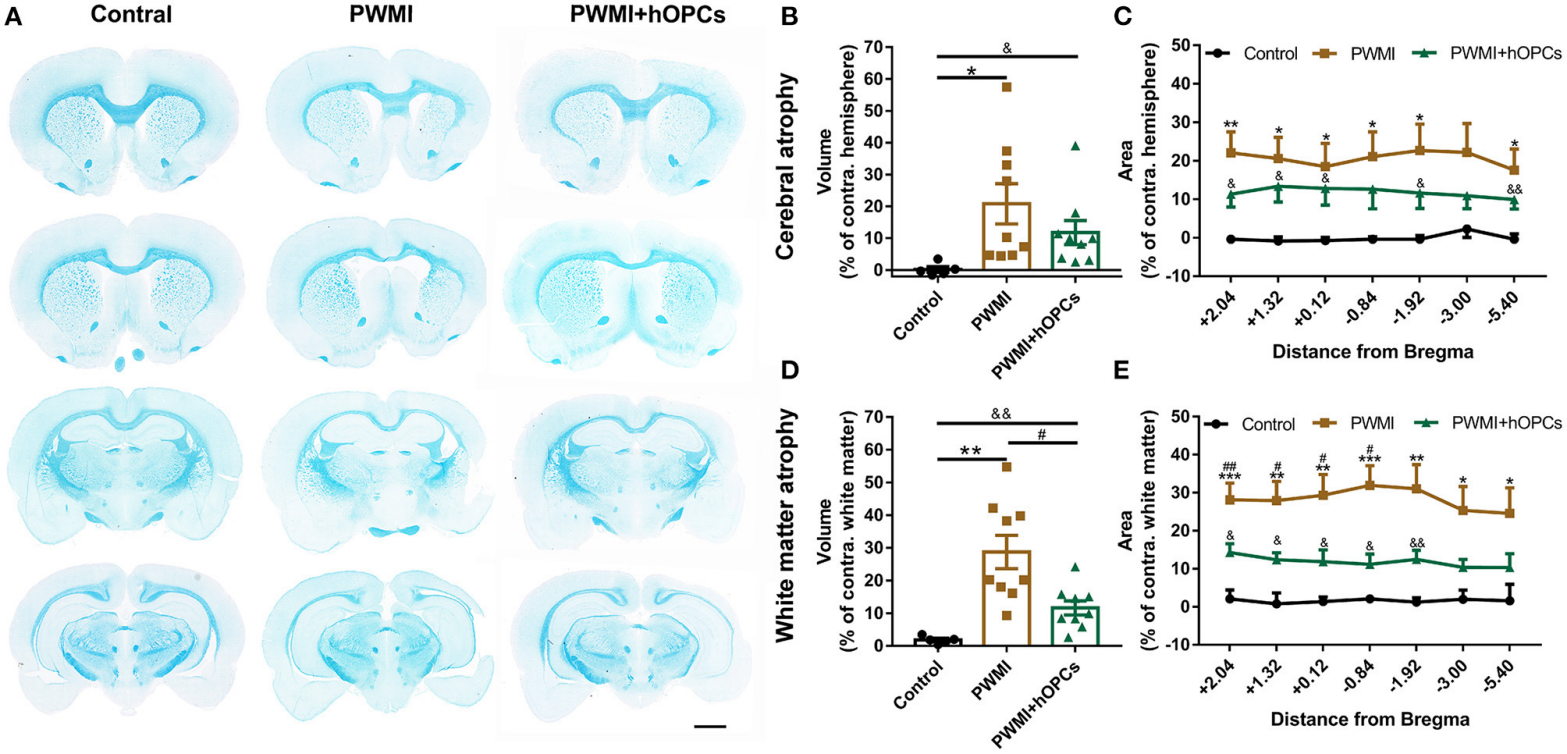
Measurement of cerebral atrophy and white matter atrophy. **(A)** Representative coronal sections stained with LFB demonstrate prominent white matter atrophy in the PWMI group and restoration following transplant. Scale bar = 2 mm. **(B,C)** Cerebral atrophy area in specific coronal sections and cerebral atrophy volume were analyzed between groups (*n* = 5 for controls, *n* = 9 for PWMI and PWMI+hOPCs; seven sections per rat; Brown-Forsythe test with Dunnett's T3 for multiple comparisons). **(D,E)** White matter atrophy area in specific coronal sections and white matter atrophy volume were compared between groups (*n* = 5 for controls, *n* = 9 for PWMI and PWMI+hOPCs; seven sections per rat; Brown-Forsythe test with Dunnett's T3 for multiple comparisons). Data are presented as mean ± SEM; PWMI vs. control: ^*^P < 0.05, ^**^P < 0.01, ^***^P < 0.001; PWMI + hOPCs vs. PWMI: ^#^*P* < 0.05, ^*##*^*P* < 0.01; PWMI + hOPCs vs. control: ^&^*P* < 0.05, ^&&^ *P* < 0.01.

At the level of gross morphology, PWMI rats showed prominent cerebral atrophy and white matter atrophy compared with control animals [cerebral atrophy, *F*_(2,20)_ = 4.578, *P* = 0.031, PWMI vs. control, *P* = 0.033; white matter atrophy, *F*_(2,20)_ = 15.629, *P* = 0.001, PWMI vs. control, *P* = 0.002; Brown-Forsythe test with Dunnett's T3 for multiple comparisons; Figures 4A,B,D]. Furthermore, cerebral atrophy along with white matter atrophy in PWMI rats was found to be relatively uniform across the rostro-caudal axis (Figures 4C,E), without specifically injured coronal levels. White matter atrophy volume was reduced in hOPC-transplanted rats (*P* = 0.03; Dunnett's T3), but the cerebral atrophy showed no significant change compared to PWMI animals. In addition, cerebral atrophy volume and white matter atrophy volume in hOPC-transplanted rats were still obvious compared to the control group (cerebral atrophy, *P* = 0.043; white matter atrophy, *P* = 0.005; Dunnett's T3; Figures 4B,D). In hOPCs-transplanted rats, the white matter of ipsilateral corpus callosum, external capsule and internal capsule was slightly sparse and structurally abnormal compared to the contralateral (see Supplementary Figure 2). These results indicated that transplantation of hOPCs can partially reserve the injured white matter tissue.

### hOPCs Alleviated Neurobehavioral Deficits in PWMI Rats

**Figure 5:**
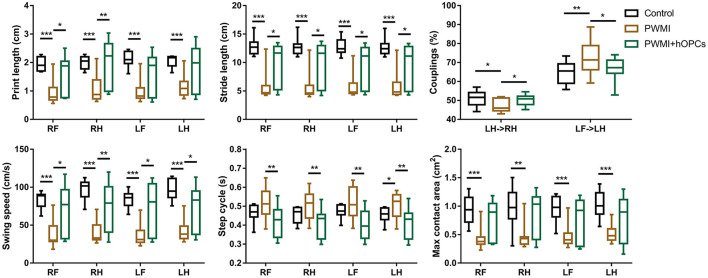
Gait analysis between groups. HI injury-induced severe alternation in print length, stride length swing speed and max contact area of each paw, as well as coupling parameters LH->RH and LF->LH. Such alternation of gait parameters was improved following hOPCs-transplantation (*n* = 10 for controls, *n* = 13 for PWMI and *n* = 12 for PWMI+hOPCs; Kruskal–Wallis non-parametric test with Mann-Whitney *U*-test for multiple comparisons). Data are presented as median (IQR); ^*^P < 0.05, ^**^P < 0.01, ^***^P < 0.001.

Gait parameters in the CatWalk test selected for analysis between groups are listed and defined in Supplementary Table 1. Compared to control animals, a severe reduction was observed in parameters that reflect locomotor function of PWMI rats: print length [RF, H_(2)_ = 11.110, *P* = 0.004, PWMI vs. control, *P* = 0.001; RH, H_(2)_ = 14.505, *P* = 0.001, PWMI vs. control, *P* < 0.001; LF, H_(2)_ = 13.009, *P* = 0.001, PWMI vs. control, *P* < 0.001; LH, H_(2)_ = 10.166, *P* = 0.006, PWMI vs. control, *P* < 0.001], stride length [RF, H_(2)_ = 15.759, *P* < 0.001, PWMI vs. control, *P* < 0.001; RH, H_(2)_ = 15.185, *P* = 0.001, PWMI vs. control, *P* < 0.001; LF, H_(2)_ = 16.520, *P* < 0.001, PWMI vs. control, *P* < 0.001; LH, H_(2)_ = 15.605, *P* < 0.001, PWMI vs. control, *P* < 0.001], swing speed [RF, H_(2)_ = 15.114, *P* = 0.001, PWMI vs. control, *P* < 0.001; RH, H_(2)_ = 17.746, *P* < 0.001, PWMI vs. control, *P* < 0.001; LF, H_(2)_ = 14.527, *P* = 0.001, PWMI vs. control, *P* < 0.001; LH, H_(2)_ = 16.258, *P* < 0.001, PWMI vs. control, *P* < 0.001] and max contact area [RF, H_(2)_ = 11.342, *P* = 0.003, PWMI vs. control, *P* < 0.001; RH, H_(2)_ = 8.694, *P* = 0.013, PWMI vs. control, *P* = 0.003; LF, H_(2)_ = 8.847, *P* = 0.012, PWMI vs. control, *P* = 0.001; LH, H_(2)_ = 11.413, *P* = 0.003, PWMI vs. control, *P* < 0.001] of each paw. Furthermore, the coupling parameters LH->RH [H_(2)_ = 3.847, *P* = 0.032, PWMI vs. control, *P* = 0.015] and LF->LH [H_(2)_ = 4.200, *P* = 0.024, PWMI vs. control, *P* = 0.01], used as a measure of inter-paw coordination, changed significantly. hOPC-transplanted animals displayed marked improvement in gait parameters including print length of right paws (RF, *P* = 0.039; RH, *P* = 0.004; LF, *P* = 0.121; LH, *P* = 0.121), stride length (RF, *P* = 0.012; RH, *P* = 0.014; LF, *P* = 0.024; LH, *P* = 0.026), swing speed (RF, *P* = 0.014; RH, *P* = 0.009; LF, *P* = 0.012; LH, *P* = 0.034), and step cycle [RF, H_(2)_ = 8.414, *P* = 0.015, PWMI+hOPCs vs. PWMI, *P* = 0.008; RH, H_(2)_ = 8.422, *P* = 0.015, PWMI+hOPCs vs. PWMI, *P* = 0.01; LF, H_(2)_ = 10.767, *P* = 0.005, PWMI+hOPCs vs. PWMI, *P* = 0.003; LH, H_(2)_ = 9.618, *P* = 0.008, PWMI+hOPCs vs. PWMI, *P* = 0.005] of each paw, as well as LH->RH (*P* = 0.045) and LF->LH (*P* = 0.044) couplings (Kruskal–Wallis non-parametric test with Mann-Whitney *U*-test for multiple comparisons; Figure 5).

**Figure 6:**
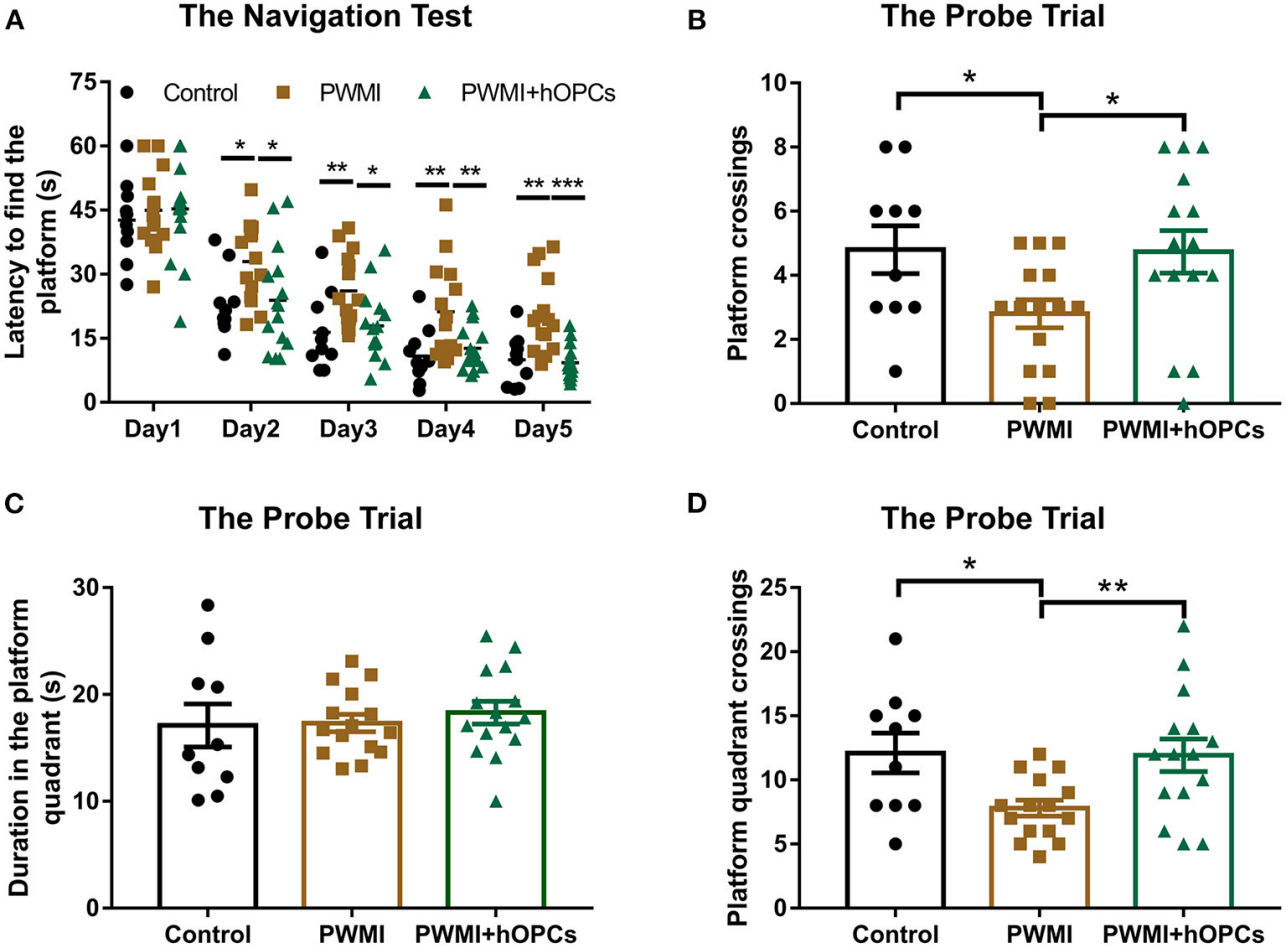
Assessment of cognitive function. **(A)** In the navigation test, increased latency to find the platform of PWMI rats were reduced to a near-normal level after hOPCs-transplantation (*n* = 10 for the control, *n* = 15 for PWMI and PWMI+hOPCs; repeated measures ANOVA, followed by LSD *post-hoc* test). **(B–D)** In the probe trial, platform crossings and platform quadrant crossings, but not duration in the platform quadrant, decreased in PWMI group and improved after hOPCs-transplantation (*n* = 10 for the control, *n* = 15 for PWMI and PWMI+hOPCs; one-way ANOVA with LSD for multiple comparisons). Data are presented as mean ± SEM; ^*^P < 0.05, ^**^P < 0.01, ^***^P < 0.001.

In the Morris water maze test, the PWMI group showed a much longer latency to find the platform compared to control animals from navigation test day 2 [Day 2, *F*_(2,37)_ = 4.221, *P* = 0.022, PWMI vs. control, *P* = 0.018; Day 3, *F*_(2,37)_ = 5.196, *P* = 0.010, PWMI vs. control, *P* = 0.008; Day 4, *F*_(2,37)_ = 6.414, *P* = 0.004, PWMI vs. control, *P* = 0.003; Day 5, *F*_(2,37)_ = 12.655, *P* = 0.001, PWMI vs. control, *P* = 0.005], suggesting impairment of spatial learning ability in these rats. By contrast, OPC-transplanted animals reduced the latency time to a near-normal level (Day 2, *P* = 0.019; Day 3, *P* = 0.012; Day 4, *P* = 0.006; Day 5, *P* = 0.001) (Repeated measures ANOVA with LSD *post-hoc* test; Figure 6A). In the probe trial, PWMI rats had much fewer platform crossings and platform quadrant crossings compared to control animals [platform crossings, *F*_(2,37)_ = 3.645, *P* = 0.036, PWMI vs. control, *P* = 0.034; platform quadrant crossings, *F*_(2,37)_ = 4.798, *P* = 0.014, PWMI vs. control, *P* = 0.016; one-way ANOVA with LSD], suggesting a spatial memory deficit. In hOPC-transplanted rats, the platform crossings and platform quadrant crossings, but not duration in the platform quadrant, were increased (platform crossings, *P* = 0.023; platform quadrant crossings, *P* = 0.01; LSD) (Figures 6B–D). Taken together, the above results indicate that locomotor and cognitive deficits of PWMI rats can be improved by engraftment of hOPCs.

## Discussion

The present study aimed to determine the fate and effectiveness of transplanted hOPCs in a rat model of PWMI. We found that hOPCs transplanted via the ventricle migrated widely and differentiated into myelin-forming OLs at 13 weeks after grafting. Transplantation of hOPCs restored myelin and rescued neurobehavioral deficits. Importantly, we first demonstrated the regenerative effect of hOPC in PWMI rats.

PWMI is a major neuropathological brain injury often seen in survivors of premature birth. In humans, the susceptibility to PWMI peaks between the gestational age of 23 and 32 weeks, which corresponds to P2 to P5 in rats with regard to white matter development (2, 17). Accordingly, OPCs and pre-OLs in neonatal rats are also vulnerable to ischemia, hypoxia, and inflammatory insults. Previous studies in neonatal rats have confirmed that ischemia (unilateral carotid artery ligation) combined with hypoxia (5–8% O_2_ for 0.5–3.5 h) induces disturbed myelination and neurological deficits (17, 28). Consistent with previous work, HI-injured rats in our study displayed obvious myelin loss and structural alterations observed by TEM and immunohistochemistry, along with extensive white matter atrophy and an enlarged lateral ventricle on the gross morphology. PWMI can cause a wide range of neurological sequelae later in life, including severe motor disabilities such as cerebral palsy and subtle consequences such as impaired perceptual abilities and cognitive function, attention problems, and psychiatric conditions (29, 30). In our study, the HI-injured neonatal rats showed decreased locomotor function, abnormal inter-paw coordination, and cognitive dysfunctions, as revealed by neurobehavioral tests. The above-mentioned neuropathological and behavioral abnormalities in HI-injured rats were similar to the key characteristics of human PWMI (2, 4).

Sensitive behavioral tests that can detect long-term deficits in PWMI rats are critical for cell replacement studies, because exogenous cells may take months to proliferate, differentiate, and integrate into the host brain (27). The CatWalk is one of the most suitable methods for the quantitative assessment of impaired gait and locomotor function in rodents, as this automated system can quickly and objectively analyze a large number of gait parameters, particularly dynamic parameters such as swing duration, and interlimb coordination, which are difficult to quantify by any other method (21). The CatWalk test has been widely used in peripheral nerve injury, spinal cord injury, and ischemic brain injury models (31–33). PWMI model is also a kind of ischemic brain injury, so we introduced it into this experiment. Based on the strength and reliability of each parameter demonstrated in previous studies (21, 22, 33), 17 parameters were selected for functional evaluation in our study, and the results showed that print length, stride length, swing speed, max contact area, and couplings were changed in PWMI rats at 12 weeks post-transplant, suggesting a long-term impairment in locomotor function and coordination. The Morris water maze, a classic test for evaluating cognitive function, revealed impaired spatial learning and memory in PWMI rats, which was consistent with the results of previous studies (13, 16). We speculate that the deficits are associated not only with white matter atrophy, which interrupts brain connection, but also with a certain extent of gray matter damage, such as damage to cortical motor areas. Although this is an animal model of WMI, gray matter cannot be completely spared.

A number of preclinical studies have confirmed the effectiveness of OPC transplantation in other demyelinating diseases (24, 34–36), but there has been little published work on hOPC therapies for PWMI. Here we showed that hNSC-derived OPCs intracerebroventricularly transplanted 4 days after injury survived for 13 weeks, disseminated mostly to injured white matter areas, and migrated along white matter tracts. The distribution preference for white matter may be due to the fact that neonatal HI injury predominantly affects the white matter, and damaged white matter has a chemotactic effect on transplanted cells. This chemotaxis is believed to be mediated by trophic factors such as stromal cell-derived factor 1α, chemokine (C-X-C motif) receptor 4, and vascular endothelial growth factor (VEGF) (37, 38). Notably, up to 82.77% of our transplanted cells differentiated into mature OLs, as detected by double immunostaining for stem121 and MBP markers. By contrast, a study by Kuai et al. revealed that embryonic stem cell (ESC)-derived OPCs displayed poor survival and limited migration ability in twitcher mice, which led to remyelination failure (39). Webber et al. showed a low differentiation rate into mature OLs of rat-derived OPCs in PWMI animals (14). Compared with the above studies, the relatively extensive migration and high-efficiency differentiation of hOPCs observed here mainly attributed to the origin and stage of transplanted cells. The hOPCs grafted in our study were induced from human fetal NSCs, different from the ESC-derived or rat-derived OPCs, and 80–90% of our hOPCs expressed PDGF-αR, A2B5, and O4, consistent with a late OPC phenotype (20), while early OPCs, expressing NG2, Olig2, and A2B5, were used by Webber et al. Secondly, contrary to the above studies, we used cyclosporine for anti-rejection to ensure the survival of exogenous cells. According to previous studies, cyclosporine treatment can reduce xenogeneic cell rejection and prolong the survival of transplanted cells (40, 41).

Importantly, our study demonstrated that transplantation of hOPCs markedly restored myelination in PWMI rats, as an increase in myelin integrity was observed via immunostaining of host MBP and ultrastructural analysis by TEM, as well as a reduction in white matter atrophy at the level of gross morphology. The mechanisms underlying myelin repair include myelin regeneration and the neuroprotective effects of the transplanted grafts. Traditionally, cell-based therapies have been introduced to replace lost or damaged cells through targeted differentiation (42). In this study, a large fraction of transplanted hOPCs differentiated into OLs and produced myelin wrapping around axons, as confirmed by co-staining and confocal scanning. This implies that the implanted hOPCs generated new myelin sheaths by cell replacement. Moreover, a growing number of studies have indicated that grafted cells can release various neurotrophic factors such as brain-derived neurotrophic factor, ciliary neurotrophic factor, VEGF, and insulin-like growth factor-1, which exert favorable effects on angiogenesis, neurogenesis, neuronal survival, and myelination (43–45). We suspected that our transplanted hOPCs can secret neuroprotective factors into the local environment, thus preventing myelin loss and promoting endogenous myelination. However, a limitation here is that we cannot separately quantify exogenous myelination and endogenous myelination induced by hOPC transplantation.

Moreover, the transplantation of hOPCs significantly improved the behavioral performance of PWMI rats. Ameliorated cognitive deficits were achieved at 12 weeks after transplantation, as observed in the Morris water maze test. Automated gait analysis revealed that transplanted hOPCs restored locomotor function and inter-paw coordination in PWMI rats. It is believed that these improved neurological functions are associated with the repair of myelin and white matter structures, which restores saltatory conduction and brain connectivity (46).

In conclusion, the present study showed that transplanted hOPCs restored the myelin of injured white matter and eventually led to neurobehavioral improvements in PWMI rats. Importantly, we first demonstrated the regenerative effect of hOPCs in PWMI animals. These results suggest that the transplantation of hOPCs may be an effective and promising therapeutic strategy for children with PWMI. Our results are encouraging for further translational studies. In addition, others in our research group have found highly expressed neurotrophic factors in hOPCs by single-cell sequencing, our follow-up research is to identify neurotrophic factors secreted by hOPCs *in vivo*, which benefit endogenous remyelination or prevent myelin loss.

## Data Availability Statement

The original contributions presented in the study are included in the article/Supplementary Material, further inquiries can be directed to the corresponding author/s.

## Ethics Statement

The studies involving human participants were reviewed and approved by Ethics Committee of the Chinese PLA General Hospital (Beijing, China). The patients/participants provided their written informed consent to participate in this study. The animal study was reviewed and approved by the Animal Ethical and Welfare Committee of the Chinese PLA General Hospital (Beijing, China).

## Author Contributions

YY, YW, and ZL designed the experiments. XW and JZ carried out HI operations and cell transplantation. ZW, DY, and QW were responsible for preparation of hOPCs. QG and KL conducted behavioral tests. SL, XW, and HZ performed histological staining and statistical analysis. XW and YY drafted the manuscript. All authors contributed to manuscript revision, read, and approved the submitted version.

## Funding

This study was supported by the National Key R&D Programme of China [grant number 2017YFA0104200], funded by Ministry of Science and Technology of the People's Republic of China.

## Conflict of Interest

The authors declare that the research was conducted in the absence of any commercial or financial relationships that could be construed as a potential conflict of interest.

## Publisher's Note

All claims expressed in this article are solely those of the authors and do not necessarily represent those of their affiliated organizations, or those of the publisher, the editors and the reviewers. Any product that may be evaluated in this article, or claim that may be made by its manufacturer, is not guaranteed or endorsed by the publisher.
