# Supplementary material for: Transplanted Human Oligodendrocyte Progenitor Cells Restore Neurobehavioral Deficits in a Rat Model of Preterm White Matter Injury
Source: Front Neurol. 2021 Nov 10;12:749244. doi: 10.3389/fneur.2021.749244 (PMC8631304; doi:10.3389/fneur.2021.749244)
Supplement: Supplementary file 1 [file Data_Sheet_1.docx]

Supplementary Material

# Supplementary Figures


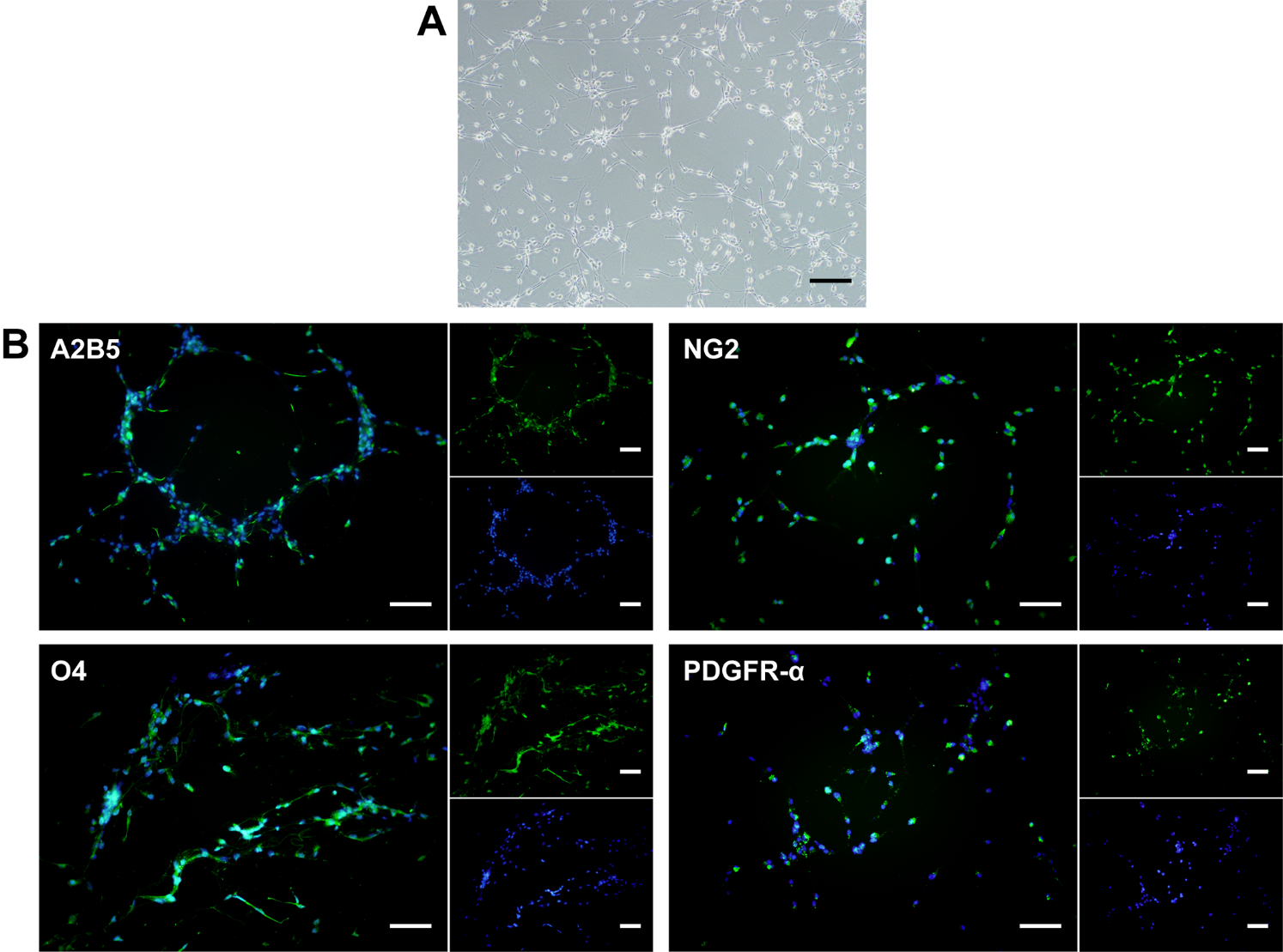


**Supplementary Figure 1.** Identification of hOPCs before transplantation. **(A)** hOPCs display a bipolar morphology under the light microscope. (B) Immunofluorescence staining of hOPC-specific markers. Cells for transplantation highly expressed A2B5, NG2, O4, and PDGFR-α. Scale bar = 100 μm.


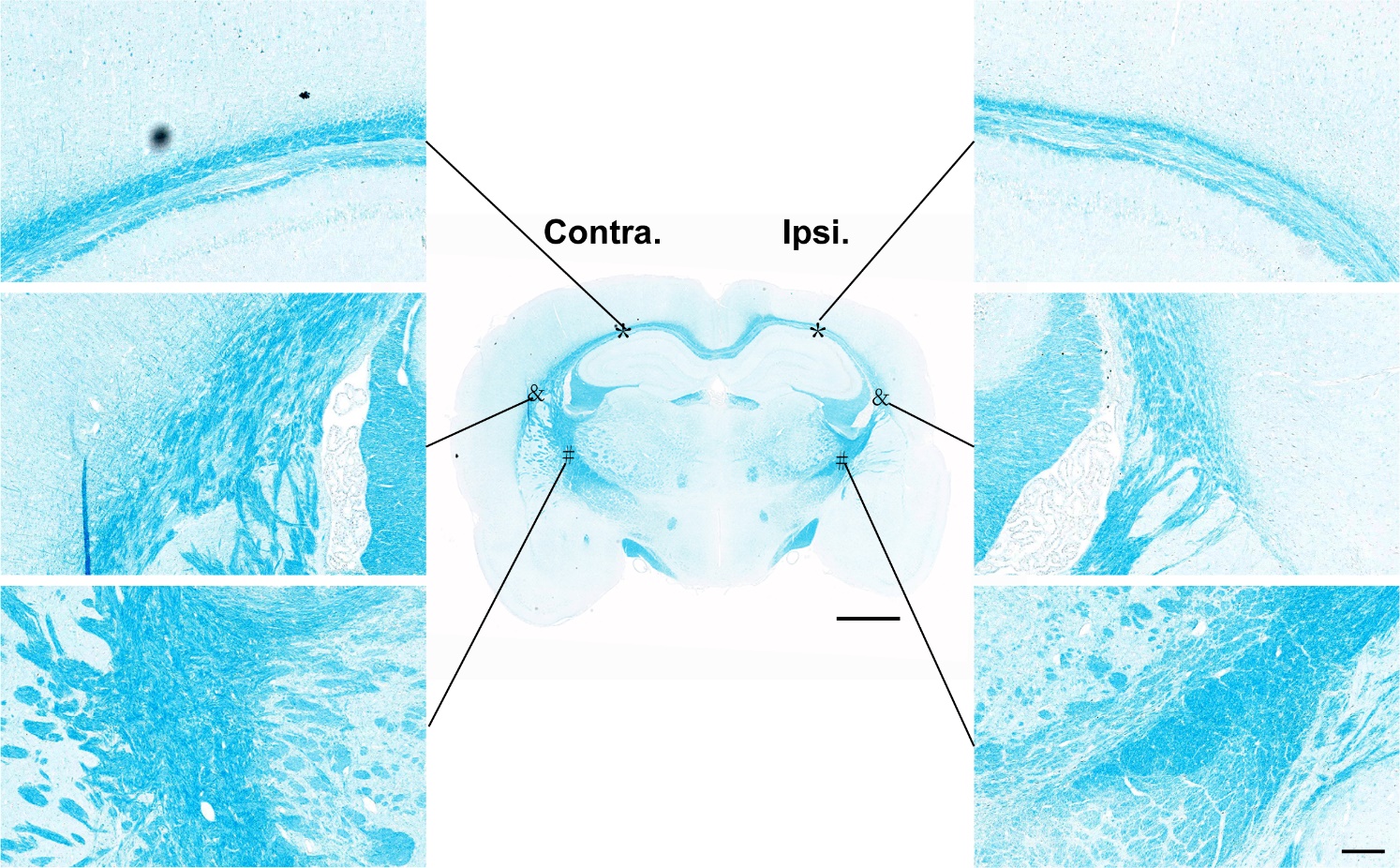


**Supplementary Figure 2.** Representative high-resolution images of the contralateral and ipsilateral white matter in hOPCs-transplanted rats. Compared with the contralateral, the white matter in ipsilateral corpus callosum (*), external capsule (&) and internal capsule (#) was slightly sparse and structurally abnormal. Scale bar represents 2000 μm in the central image and 200 μm in surrounding images.

# Supplementary Tables

| **Parameter** | **Definition** |
| --- | --- |
| Print position (cm) | Distance from the former forepaw position to the consecutive hindpaw position |
| Print size (cm^2^) | The surface area of the complete print |
| Print length (cm) | Length (horizontal direction) of the complete print |
| Base of support (cm) | Average width between either the front paws or the hind paws |
| Intensity (a.u.) | Average print intensity at the moment of maximal contact expressed as arbitrary |
| Max contact area (cm^2^) | The size of the print area at maximal contact |
| Run duration (s) | Time duration of the entire run |
| Body speed (cm/s) | Distance that an animal walked per second |
| Stride length (cm) | Distance between successive placements of the same paw during maximal contact |
| Step cycle (s) | Duration between two successive initiations of stances (=stance duration + swing duration) |
| Stance duration (s) | Duration of the stance phase |
| Swing duration (s) | Duration of the swing phase |
| Swing speed (cm/s) | Stride length over the swing duration |
| Duty cycle (%) | Ratio of the stance duration to the step cycle duration |
| Support (% time standing) | Percentage of time two paws are in simultaneous contact with the floor |
| Couplings (%) | Percentage of time the target paw takes to step in relation to the step cycle of the anchor paw |
| Regularity index (%) | The number of normal step sequence patterns relative to the total number of paw placements |

**Supplementary Table 1.** Definition of CatWalk gait parameters. Seventeen parameters with high reliability were selected for functional evaluation in our study.
